# Characterization of US Hospital Advertising and Association With Hospital Performance, 2008-2016

**DOI:** 10.1001/jamanetworkopen.2021.15342

**Published:** 2021-07-02

**Authors:** Chima D. Ndumele, Michael S. Cohen, Muriel Solberg, Anthony Lollo, Jacob Wallace

**Affiliations:** 1 Department of Health Policy and Management, Yale School of Public Health, New Haven, Connecticut; 2 Congressional Budget Office, Washington, DC; 3 Yale School of Medicine, New Haven, Connecticut

## Abstract

**Importance:**

Hospital advertising has been touted as a tool to improve consumer decision-making, but little is known about its association with objective measures of hospital quality.

**Objective:**

To document recent trends in hospital advertising in the US and examine the association between concurrent measures of hospital advertising and quality.

**Design, Setting, and Participants:**

Retrospective cross-sectional study of all general acute care hospitals operating in the US between January 2008 and December 2016. Data were analyzed from December 6, 2019, to July 15, 2020.

**Exposure:**

Annualized advertising spending for each hospital as measured by a market research firm.

**Main Outcomes and Measures:**

Four composites of hospital performance from the Centers for Medicare & Medicaid Services Hospital Compare database were used: risk-standardized mortality rate, risk-standardized readmission rate, Consumer Assessment of Healthcare Providers & Systems (CAHPS) Overall Patient Experience Rating (scale of 1-5; higher scores indicate a more positive patient experience rating), and overall 5-star rating. Linear models adjusted for hospital bed size, hospital revenue, and geographic census region.

**Results:**

The study sample included, on average, 4569 general acute care hospitals per year between 2008 and 2016. During this time, approximately half of acute care hospitals (2239 of 4569 [49%]) advertised their services to consumers and spent a total of $3.39 billion. Relative to hospitals that never advertised, advertising hospitals were more likely to be nonprofit facilities (mean [SD], 66% [47%] vs 51% [50%]; *P* < .001), had larger bed sizes (mean [SD], 234.3 [210.7] beds vs 84.8 [110.6] beds; *P* < .001), and had higher net incomes (mean [SD], $17 800 000 [$49 000 000] vs $134 099 [$51 600 000]; *P* < .001). There was no observed association between hospital advertising and performance. For example, hospitals that advertised had a mean (SD) CAHPS 5-star rating of 3.2 (0.9) stars compared with 3.3 (1.0) stars among hospitals that did not advertise, an insignificant difference (*P* = .92). We observed no difference in performance between advertising and nonadvertising hospitals in 30-day readmission rates (mean [SD], 15.5% [0.8%] vs 15.6% [1.0%]; *P* = .25), mortality rates (mean [SD], 12.7% [4.0%] vs 12.0% [4.1%]; *P* = .46), and overall 5-star hospital ratings (mean [SD], 3.1 [0.8] stars vs 3.0 [0.9] stars; *P* = .50). A significant difference was observed in adjusted mortality rates across terciles of advertising spending, with lower mortality rates for the hospitals with higher ad spending (2016, mean [SD] mortality composite for hospitals in the highest tercile, 11.2% [4.2%] vs hospitals in the middle tercile, 12.0% [3.8%], and for hospitals in the lowest tercile, 12.7% [4.1%]; *P* = .003).

**Conclusions and Relevance:**

The results of this cross-sectional study suggest that the amount hospitals spent on direct-to-consumer advertising was not associated with publicly reported measures of hospital quality; instead, hospital advertising spending was higher for financially stable hospitals with higher net incomes.

## Key Points

**Question:**

Are higher-performing hospitals more likely to advertise their services directly to consumers?

**Findings:**

In this cross-sectional study of more than 4500 acute care hospitals per year, almost half advertised their services to consumers, yet there was no clear association between advertising and objective performance measures. Hospitals that advertised were more likely to have more net assets and income.

**Meaning:**

The results of this cross-sectional study suggest that advertising is not an effective means to inform consumers about quality; instead, it may mislead consumers and undermine, not complement, public reporting efforts.

## Teaser

This cross-sectional study uses data from the Centers for Medicare & Medicaid Services to assess whether higher-performing hospitals are more likely to advertise their services directly to consumers.

## Introduction

Direct-to-consumer advertising (DTC) is a common feature of competitive health care markets, with a reported $29 billion spent on health care advertising in the US in 2016.^1,2,3,4^ In theory, DTC advertising allows organizations to inform consumers about the breadth and quality of services they offer, increasing awareness and improving decision-making.^5,6^ In practice, however, DTC advertising in health care has been found to include misleading or inaccurate statements, perhaps owing to limited oversight, raising concerns that advertising may be a mechanism for health care organizations to increase the demand for potentially unneeded services.^7,8,9,10^ Hospital advertising presents a challenge for regulators who must balance the potential benefits of informing consumer choice against the need for guardrails to protect unsuspecting patients. Prior work has shown that hospital advertising influences consumer choices, but it is unknown whether it sways consumers to make better decisions.^11,12,13^

Hospital advertising was discouraged in the US until the early 1980s, when policymakers began to embrace it as part of a broader strategy to inform consumers about the differences in quality across health care facilities.^10^ The hope was that higher quality hospitals would use advertising to market their superior performance to consumers, increase the demand for high-quality relative to low-quality hospitals, and, ultimately, increase the incentives for all hospitals to invest in quality improvement.^14^ Over time, however, policy observers have raised concerns that advertising may instead be used to increase the demand for all hospitals, not just high-quality facilities, because of the difficulty consumers have in judging the quality claims made by hospitals.^15^ Moreover, as a result of limited regulation, advertisements may be designed to mislead consumers.^16^ For example, prior studies have found that cancer center advertisements were more likely to promote the benefits of treatments rather than their potential risks; in addition, cancer center advertisements tended to be overly optimistic in relaying prognoses.^15,17,18^ To our knowledge, few studies have examined the use of advertising by general hospitals and, in particular, whether it is more common among high-quality hospitals.^1,18^

Owing to the lack of oversight and the difficulty consumers have validating claims made by hospitals in their advertisements, understanding whether high-quality hospitals are responsible for most of the DTC hospital advertising is a critical policy question. Using data from a market research firm, we examined trends in hospital advertising in the US, documented the characteristics of the hospitals that advertise, and assessed differences in publicly reported quality for hospitals that do and do not advertise.

## Methods

### Overview

Our cross-sectional study analyzed annual spending on advertising by general acute care hospitals operating in the US from January 2008 to December 2016, and assessed the association between hospital advertising and concurrent measures of hospital performance. This study was deemed exempt by the Yale University institutional review board because it only included the analysis of secondary data. In reporting the methods and results, we followed the Strengthening the Reporting of Observational Studies in Epidemiology (STROBE) reporting guidelines for cross-sectional studies.

### Sources of Data and Study Population

Data on annual advertising expenditures were obtained from Kantar Media, a market research firm that tracks organizations’ advertising expenditures at the designated market area (DMA)–by-month level. The data have been used in previous studies examining advertising in health care.^3,9,19^ Designated market areas consist of groups of counties surrounding metropolitan areas and were developed by Nielsen Holdings (a data and market measurement company) to track television viewership. Media advertising is typically purchased at the DMA level. The advertising data include monthly spending by each entity, the method of advertising (eg, newspaper), and the DMA where the advertisement was run. The study period ranged from 2008 to 2016, as 2008 was the first year that Kantar data covered all DMAs in the US. We included all spending for hospitals with at least $25 000 in annual advertising spending in 1 year of our sample, which represented 98% of hospital advertising spending in the data set. If hospitals never reached annual spending of at least $25 000 during the study period, they were classified as a hospital without substantial advertising spending. To identify general acute care hospitals, we matched Kantar advertising data to Centers for Medicare & Medicaid Services (CMS) public use files, which provided the list of all hospitals that contract with the CMS.^20^ Details on our matching process are in eAppendix 1 in the Supplement.

Data on annual hospital performance are from the CMS Hospital Compare website.^21^ Hospital data submissions for Hospital Compare are from Medicare claims data, surveys conducted by hospitals, and medical records that are submitted to CMS Clinical Data Warehouse and periodically audited by CMS to ensure accuracy. From these data, we assessed 3 measures of domain-specific performance: risk-standardized hospital readmission rates, patient experience scores from the Hospital Consumer Assessment of Healthcare Providers and Systems, and the overall performance rating for each hospital. A fourth performance measure, a mortality rate composite, was constructed using a weighted average of hospital-level mortality rates for 6 conditions or procedures reported by CMS: congestive heart failure, acute myocardial infarction, pneumonia, stroke, chronic obstructive pulmonary disease, or coronary artery bypass grafting. Additional details on the composite measures are described in eAppendix 2 in the Supplement and also available elsewhere.^22^

Data on each hospital’s organizational structure were obtained from CMS Hospital Cost Reports for the years 2008 to 2016.^23^ These data include information on facility characteristics, use of services, and revenue sources for the hospitals that bill Medicare. These data were linked to data from the Dartmouth Atlas that matched each hospital to a hospital referral region (HRR).^24^ The final analytic sample included, on average, 4569 general acute care hospitals per year that contracted with CMS during the period 2008 to 2016. Descriptive data for advertising trends and hospital characteristics are provided for all study years 2008 to 2016. Because Hospital Compare only provided composite measures for all 4 domains in 2016, we limited analyses of the association between hospital spending on advertising and concurrent measures of hospital performance to that year.

### Variables

The primary outcomes (dependent variables) were composite measures of hospital performance in 4 domains: risk-standardized mortality rate, risk-standardized readmission rate, patient experience score, and overall 5-star composite rating. The 5-star composite scores were publicly reported on the CMS Hospital Compare website starting in 2016 and were constructed using latent variable models (eAppendix 2 in the Supplement). Composite scores were also risk adjusted by CMS to account for the differences in case mix across hospitals.

Annual advertising expenditure was calculated using Kantar Media data as described previously. If the listed advertiser was a hospital system (eg, Partners Healthcare), total advertising dollars were apportioned equally to all hospitals affiliated with that system within the DMA where the advertising took place. If the listed advertiser was a specific hospital, spending was attributed to the hospital of record in the Kantar Media advertising data set. In primary analyses, hospitals were grouped on a yearly basis into 1 of 4 categories: those without substantial advertising spending (<$25 000) and, for those with substantial advertising spending, into terciles based on the amount spent on advertising in that year.

### Statistical Analysis

The primary analysis was conducted from December 6, 2019, to July 15, 2020, and examined the extent of, and trends in, hospital advertising in the US and the association between hospital advertising and hospital quality. First, we assessed total hospital spending on DTC advertising, and the percentage of hospitals that advertise, for each year of our study period. To examine the hospital characteristics associated with hospital advertising expenditure, each hospital was assigned to 1 of the 4 advertising spending categories annually: no advertising, low ad spending, medium ad spending, or high ad spending. This approach allowed hospitals to move across advertising spending categories from year to year, which was something we observed in the data. If necessary, missing information on hospital characteristics was imputed based on the most recent available data.

The analyses explored the concurrent association between hospital advertising and publicly available measures of hospital performance. First, we constructed linear regression models examining the association between performance in each of the 4 domains and categories of hospital advertising expenditure in 2016. These models adjusted for hospital bed size, net income, and the geographic census region in which the hospital operated.

Second, we estimated ordered logistic and linear regression models to assess the association between hospital performance and the amount spent on advertising within the set of hospitals that advertised. Third, because the association between advertising and performance at the national level may not be representative of the dynamic in each market, we examined how the mean performance across the 4 domains differed between advertisers and nonadvertisers for each HRR.

Additional analyses tested the sensitivity of results to alternative specifications or sample restrictions. First, we assessed the association between hospital advertising and quality in 2013 to 2015 (using the narrower set of composites available) to ensure that our 2016 results were not outliers. Second, we limited HRR-level analyses to HRRs with at least 5 hospitals with publicly reported performance data. Third, we assessed the association between continuous measures of advertising spending and hospital quality using linear models and visualized the association using binned scatterplots. Fourth, we assessed the robustness of our results to excluding hospital years with missing data (rather than imputing). Analyses were conducted using Stata/SE, version 14.1 (StataCorp LLC), and results from all statistical tests are reported with *P* values derived from 2-tailed tests of significance and robust SEs (at *P* < .05).

## Results

### Trends in Hospital Advertising

**Figure 1.**
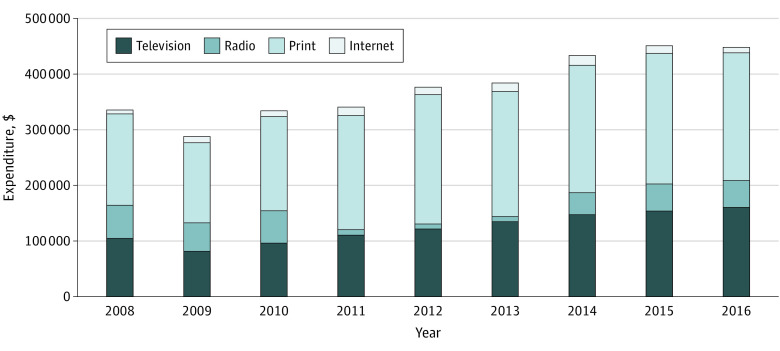
Mean Hospital Expenditure for Advertisement Via Television, Radio, Print, and Internet The print category includes newspaper, magazines, and outdoor advertisements. Expenditure is in US dollars.

The study sample included, on average, 4569 general acute care hospitals per year between 2008 and 2016. During this time, approximately half of acute care hospitals (2239 of 4569 [49%]) spent at least $25 000 in DTC advertising in at least 1 year during the study period, ranging from a low of 1716 of 4423 hospitals (38.8%) in 2008 to 2001 of 4579 hospitals (43.7%) in 2016 (eTable 1 in the Supplement). Among the hospitals that advertised, the mean (SD) hospital expenditure was $197 591 ($442 507) annually, for a total of $3.39 billion spent on DTC hospital advertising over the study period. The largest single medium for advertising spending was television (TV) advertising (32.5% of total advertising expenditures), which increased from $104 865 000 of $335 083 000 (31.3%) in total hospital expenditures in 2008 to $160 726 000 of $447 745 000 (35.9%) in 2016 (Figure 1). Relative to hospitals that never advertised, hospitals that did advertise were more likely to be nonprofit facilities (mean [SD], 66% [47%] vs 51% [50%]; *P* < .001), had larger bed sizes (mean [SD], 234.3 [210.7] beds vs 84.8 [110.6] beds; *P* < .001), and had higher net incomes (mean [SD], $17 800 000 [$49 000 000] vs $134 099 [$51 600 000]; *P* < .001) (Table 1). Among hospitals that advertised, those in the highest tercile compared with those in the bottom tercile of advertising spending had more assets (mean [SD], $174 079 325 [$269 372 738] vs $69 692 515 [$144 073 654]) and higher levels of occupancy (mean [SD], 65% [17%] vs 52% [19%]), and were more likely to be major teaching hospitals (23% [42%] vs 6% [24%]) than those in in the bottom tercile (*P* < .05 for all comparisons). Imputation for missing values did not meaningfully change our results (eTable 2 in the Supplement).

**Table 1.** Characteristics of Advertising and Nonadvertising Hospitals

Variable	Tercile of advertising spending, mean (SD)			
	No advertising	Bottom tercile	Middle tercile	Highest tercile
No. of hospital-years	23 579	5723	5714	5701
Advertising expenditure per year, $	0 (0)	14 076 (10 346)	73 464 (28 889)	506 224 (665 760)
Net income, $	134 099 (51 582 888)	9 909 325 (43 758 312)	13 945 793 (38 494 245)	29 610 067 (59 832 911)
Total current assets, $	30 973 254 (83 241 481)	69 692 515 (144 073 654)	84 534 729 (154 358 617)	174 079 325 (269 372 738)
All inpatient discharges, No.	3619.3 (5859.6)	7970.8 (7980.3)	10 127.9 (9439.1)	17 328.5 (13 494.3)
Beds, No.	84.8 (110.2)	165.65 (149.4)	202.98 (174.5)	335.0 (254.4)
Bed-days available (fully staffed), No.	30 535.0 (40 150.1)	59 800.9 (54 194.4)	73 589.9 (63 365.7)	121 558.2 (92 811.6)
Occupancy, %	41 (20)	52 (19)	56 (18)	65 (17)
Employee full-time equivalents, No.	499.9 (821.4)	974.6 (1219.4)	1263.9 (1429.2)	2321.9 (2363.9)
Medicaid inpatient-day share, %^a^	16 (13)	19 (13)	19 (12)	21 (12)
Major teaching hospital, %^b^	3 (16)	6 (24)	9 (29)	23 (42)
Nonprofit, %	51 (50)	63 (48)	64 (48)	72 (45)
For profit, %	19 (39)	24 (43)	25 (43)	17 (37)
Public, %	29 (46)	13 (33)	11 (31)	12 (32)

^a^ Includes health maintenance organizations.

^b^ Interns and residents to bed ratio greater than 0.2.

### Hospital Advertising and Performance

We did not find evidence of a consistent association between hospital spending on advertising and publicly reported measures of hospital quality (Table 2). In adjusted models, the estimated differences in performance between advertising and nonadvertising hospitals were small and insignificant for the 4 domains of care we examined. For example, hospitals that advertised had a mean (SD) CAHPS 5-star rating of 3.2 (0.9) stars vs 3.3 (1.0) stars among hospitals that did not advertise, an insignificant difference (*P* = .92). There were no significant differences between advertising and nonadvertising hospitals for the other 3 measures of performance. There were also few discernible trends in performance among advertising hospitals across terciles of advertising spending (Table 2). For hospitals that spent a mean (SD) of $14 000 ($10 000) (lowest tercile), $73 000 ($29 000) (median tercile), or $506 000 ($666 000) (highest tercile), mean (SD) CAHPS performance scores were 3.2 (0.9) stars, 3.3 (0.9) stars, and 3.2 (0.9) stars, respectively (*P* = .24). We observed no difference in performance between advertising and nonadvertising hospitals in 30-day readmission rates (mean [SD], 15.5% [0.8%] vs 15.6% [1.0%]; *P* = .25), mortality rates (mean [SD], 12.7% [4.0%] vs 12.0% [4.1%]; *P* = .46), and overall 5-star hospital ratings (mean [SD], 3.1 [0.8] stars vs 3.0 [0.9] stars; *P* = .50) (Table 2). We were also unable to reject the null of no differences across terciles of hospital advertising spending in performance on the overall 5-star hospital ratings (mean [SD], lowest tercile, 3.0 [0.9] stars; median tercile, 3.0 [0.9] stars; highest tercile, 2.9 [0.9] stars; *P* = .71) and on hospital readmission rate (mean [SD], lowest tercile, 15.6% [0.9%]; median tercile, 15.6% [1.0%]; highest tercile, 15.7% [1.0%]; *P* = .59).

**Table 2.** Adjusted Mean Differences in Performance^a^

Variable	Mean (SD)						
	Differences between hospitals that do and do not advertise			Differences between advertising hospitals based on intensity of advertising by tercile			
	Do not advertise	Advertise	*P* value^b^	Bottom tercile	Middle tercile	Highest tercile	*P* value^c^
Overall 5-star rating, stars	3.1 (0.8)	3.0 (0.9)	.50	3.0 (0.9)	3.0 (0.9)	2.9 (0.9)	.71
CAHPS 5-star rating, stars	3.3 (1.0)	3.2 (0.9)	.92	3.2 (0.9)	3.3 (0.9)	3.2 (0.9)	.24
Mortality composite, %	12.7 (4.0)	12.0 (4.1)	.46	12.7 (4.1)	12.0 (3.8)	11.2 (4.2)	.003
Overall readmission rate, %	15.5 (0.8)	15.6 (1.0)	.25	15.6 (0.9)	15.6 (1.0)	15.7 (1.0)	.59

Abbreviation: CAHPS, Consumer Assessment of Healthcare Providers & Systems.

^a^ Results from 2016 spending and performance data.

^b^ Column reflects *P* value from *t* test on the coefficient for a dummy variable indicating any advertising spending in a model that controls for hospital beds, net income, and census region. Ordinal logit model was used for CAHPS 5-star measure and linear regression model was used for mortality and readmissions measures.

^c^ Column reflects *P* value from joint *F* test of equality of spending category coefficients from model that controls for number of hospital beds, net income, and census region. Ordinal logit model was used for CAHPS 5-star measure and linear regression model was used for mortality and readmissions measures.

We did, however, observe a significant difference in adjusted mortality rates across terciles of advertising spending, with lower mortality rates for the hospitals with higher ad spending (2016, mean [SD] mortality composite for the hospitals in the highest tercile, 11.2% [4.2%], for hospitals in the middle tercile, 12.0% [3.8%], and for hospitals in the lowest tercile, 12.7% [4.1%]; *P* = .003), indicating that there are dimensions in which advertising may communicate better performance. Our findings were qualitatively similar when we assessed hospital advertising and quality for 2013 to 2015 (eTable 3 in the Supplement) and when modeling the linear association between continuous measures of hospital quality and advertising spending (eFigure and eTable 4 in the Supplement).

**Figure 2.**
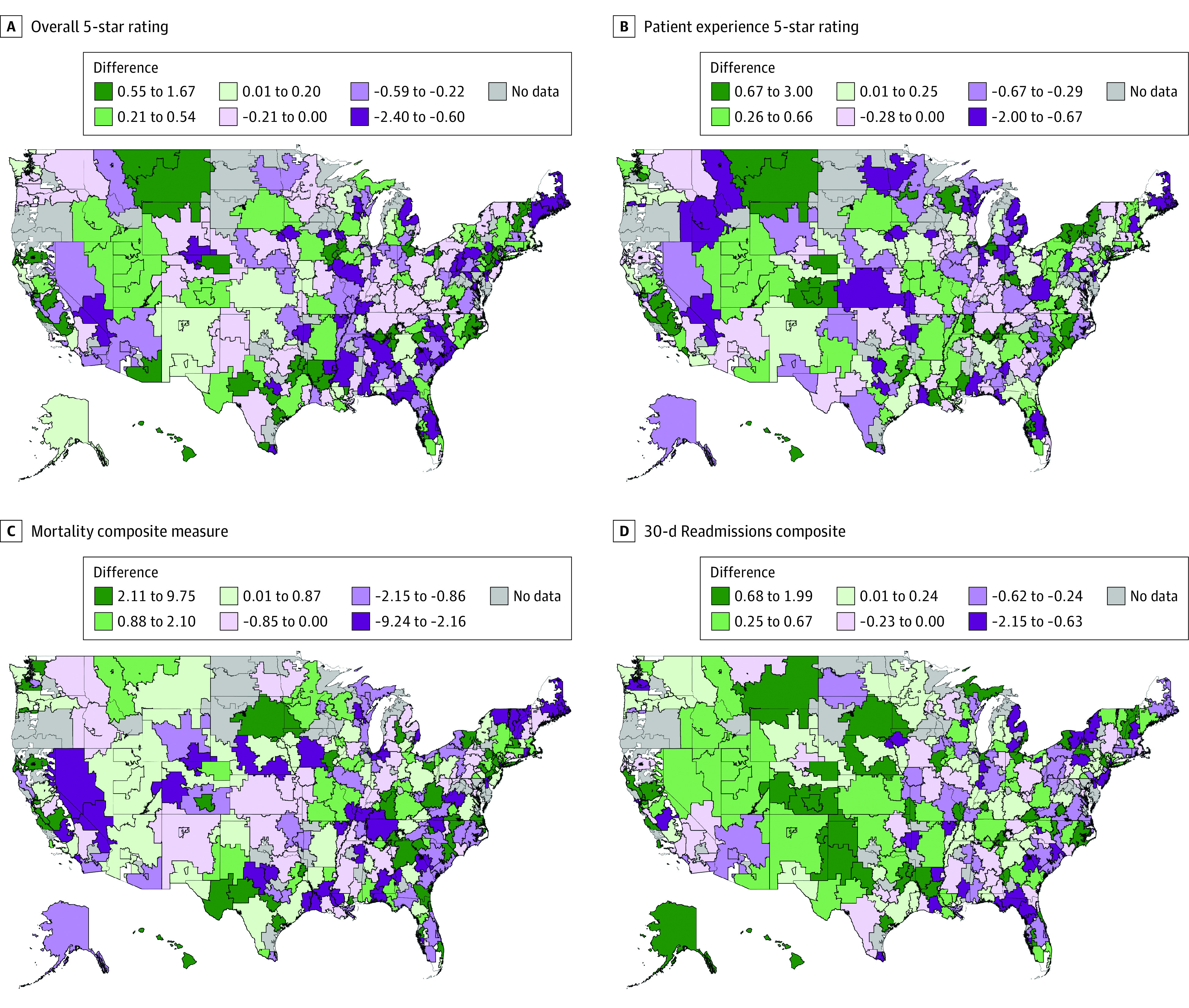
Mean Performance Between Hospital Types That Vary by Hospital Referral Region (HRR) and Domains of Care HRRs with shades of green indicate that average performance is better among hospitals that advertise; purple indicates that average performance is better among nonadvertising hospitals.

While adjusted models showed no difference in performance between advertising and nonadvertising hospitals at the national level, we found differences in mean performance between hospital types that varied by HRR and domains of care (Figure 2). Because of the large number of HRRs, rather than conduct many statistical tests of the differences in mean performance between advertisers and nonadvertisers within each HRR, we compared the mean performance of advertising and nonadvertising hospitals in each HRR. In slightly more than half of HRRs, nonadvertising hospitals had better average performance relative to advertising hospitals for overall 5-star ratings (51% vs 49%), CAHPS 5-star ratings (52% vs 48%), and mortality rates (51% vs 49%), whereas slightly fewer than half had better average readmission rates (46% vs 54%) (eTable 5 in the Supplement). These findings were robust when the analysis was limited to HRRs with 5 or more hospitals with performance data: overall 5-star ratings (51% vs 49%), CAHPS 5-star ratings (52% vs 48%), mortality rates (52% vs 48%), and average readmission rates (46% vs 54%) (eTable 6 in the Supplement).

## Discussion

Approximately half of the acute care hospitals in the US engaged in advertising between 2008 and 2016, spending $3.39 billion over that period. We did not find evidence that the hospitals that advertised were those that performed better on publicly reported hospital quality measures. Advertising hospitals reported lower average risk-standardized mortality rates than nonadvertising hospitals in approximately 49% of hospital referral regions, indicating that a person choosing a hospital based solely of whether they advertised would have, on average, no better odds of picking a higher-performing hospital than if they were choosing at random from the set of hospitals within their HRR. The results were similar for our other outcome measures. Moreover, among the hospitals that did advertise, we found little association between the amount spent on advertising and quality. Rather, we found evidence that the more financially stable hospitals—which tended to be teaching hospitals with more assets and higher levels of occupancy—did the most advertising. To the extent that hospital advertising increases demand, this suggests that DTC advertising could harm hospitals struggling financially rather than reward high-quality hospitals as intended.

This study investigated an emerging challenge in health care—how should consumers identify the high-quality hospitals in their market? One approach has been to publicly report on hospital quality, wherein governments and other community organizations invest resources in measuring hospital performance and make that information publicly available.^25,26^ However, this approach has generally led to mixed results owing to a lack of consumer engagement and the difficulty consumers have interpreting performance data.^25,26,27^ Policy observers hoped that DTC advertising offered another avenue by which organizations could communicate their performance to consumers, perhaps doing so in a manner that was more engaging or provided more distilled signals of quality than what was publicly reported.^10^ However, our results suggest that there was not a strong association between the quality of a hospital and the extent to which that hospital engages in DTC advertising, potentially undermining this goal. One concern is that the highest-quality hospitals cannot signal their true quality because consumers cannot distinguish their claims from those made by lower-performing hospitals.^12^ A second concern with hospital advertising is that the messages from the health care facilities may conflict with information from objective sources and, rather than complement public reporting as intended, serve to undermine it.

Hospital advertising has substantial consequences for the demand for a hospital’s services.^11^ For example, 1 prior study found that a $1000 increase in hospital advertising spending was associated with an increase of 10.1 patients per month. Other studies have found that advertising by hospitals can be effective at increasing both a hospital’s market share and the ratings provided by consumers.^6^ In some areas, for-profit hospitals have largely been responsible for the rise in hospital advertising, which raises additional questions about the intersection between profit motive and socially beneficial advertising.^28^ Although we do not find that advertising nationwide is propelled by for-profit entities, if the most financially stable hospitals advertise (rather than the highest-performing hospitals) and if consumers cannot distinguish between the competing claims, it is unclear how the substantial resources devoted to DTC hospital advertising could improve consumer choices. Rather, DTC advertising may channel consumers to hospitals with more slack resources, potentially limiting revenue for community or safety net hospitals responsible for treating the most vulnerable patient populations. Unlocking the potential of competitive health care markets will require greater concordance between what is communicated to patients and what occurs in practice.

Some policymakers have proposed bans on hospital advertising, but these efforts have generally failed.^29,30^ An 1957 article published in *JAMA* titled “Principles of Medical Ethics” deemed the practice of soliciting patients to be unethical, yet this stigma has dissipated over time.^31^ Regulation of this market has proved challenging, but more work is needed to determine which organizations would be best suited (and resourced) to provide oversight of the complex mediums in which hospitals advertise.^32^ Identifying the best path forward requires further research into the link between advertising, health care demand, and patient outcomes.

### Limitations

There were several limitations to this study. First, our study was only designed to assess cross-sectional differences in quality among the selection of hospitals who do or do not engage in DTC advertising, but hospital advertising may signal improving hospital performance that our study design was unequipped to capture. Second, although our study examined hospital performance across several domains and measures, our set of performance measures was not exhaustive and only included assessment on fee-for-service Medicare enrollees. It is also possible that our conclusions about the link between hospital performance and advertising may not generalize to measures of performance that we did not include in the study. Third, we did not have detailed information on the specific content of the advertising; thus, some hospitals may have advertised in targeted areas of specialization that may not have been captured in our analysis.

## Conclusions

The results of this cross-sectional study indicate that half of general acute care hospitals in the US advertised their services between 2008 and 2016. Results suggest that there was not an association between publicly reported measures of hospital quality and hospital spending on DTC advertising; instead, hospital advertising was higher for financially stable hospitals with more slack resources.

## Notes

Supplement. **eAppendix 1.** Matching Advertising Data to CMS Hospital Information **eAppendix 2.** Details on Composite Scores **eTable 1.** Percentage of Advertising and Nonadvertising Hospitals (2008-2016) **eTable 2.** Characteristics of Advertising and Nonadvertising Hospitals **eTable 3.** Adjusted Mean Differences in Performance by Year (2013-2015) **eFigure.** Relationship Between Ventile of Spending and Performance Measures **eTable 4.** Relationship Between Ad Spending and Quality Measures **eTable 5.** Comparison of Hospital Performance Between Advertisers and Nonadvertisers Within All HRRs **eTable 6.** Comparison of Hospital Performance Between Advertisers and Nonadvertisers Within HRRs in Five or More Hospitals eReferences
