# Supplementary material for: Characterization of US Hospital Advertising and Association With Hospital Performance, 2008-2016
Source: JAMA Netw Open. 2021 Jul 2;4(7):e2115342. doi: 10.1001/jamanetworkopen.2021.15342 (PMC8254134; doi:10.1001/jamanetworkopen.2021.15342)
Supplement: Supplement. — eAppendix 1. Matching Advertising Data to CMS Hospital Information eAppendix 2. Details on Composite Scores eTable 1. Percentage of Advertising and Nonadvertising Hospitals (2008-2016) eTable 2. Characteristics of Advertising and Nonadvertising Hospitals eTable 3. Adjusted Mean Differences in Performance by Year (2013-2015) eFigure. Relationship Between Ventile of Spending and Performance Measures eTable 4. Relationship Between Ad Spending and Quality Measures eTable 5. Comparison of Hospital Performance Between Advertisers and Nonadvertisers Within All HRRs eTable 6. Comparison of Hospital Performance Between Advertisers and Nonadvertisers Within HRRs in Five or More Hospitals eReferences [file jamanetwopen-e2115342-s001.pdf]

Supplementary Online Content

Ndumele CD, Cohen MS, Solberg M, Lollo A, Wallace J. Characterization of US hospital advertising and association with hospital performance, 2008-2016. *JAMA Netw Open*. 2021;4(7):e2115342. doi:10.1001/jamanetworkopen.2021.15342

eAppendix 1. Matching Advertising Data to CMS Hospital Information

eAppendix 2. Details on Composite Scores

eTable 1. Percentage of Advertising and Nonadvertising Hospitals (2008-2016)

eTable 2. Characteristics of Advertising and Nonadvertising Hospitals

eTable 3. Adjusted Mean Differences in Performance By Year (2013-2015)

eFigure. Relationship Between Ventile of Spending and Performance Measures

eTable 4. Relationship Between Ad Spending and Quality Measures

eTable 5. Comparison of Hospital Performance Between Advertisers and Nonadvertisers Within All HRRs

eTable 6. Comparison of Hospital Performance Between Advertisers and Nonadvertisers Within HRRs in Five or More Hospitals

eReferences

This supplementary material has been provided by the authors to give readers additional information about their work.

© 2021 Ndumele CD et al. *JAMA Network Open.*

eAppendix 1. Matching Advertising Data to CMS Hospital Information

Data on advertising expenditures came from Kantar Media, a market research firm which tracks organizations' advertising expenditures at the Designated Market Area (DMAs) level. DMAs typically consist of a group of counties surrounding a metro area and were developed by Nielsen to track television viewership. DMAs also represent the unit at which media advertising is typically purchased, such that all households within a DMA have the potential to be exposed to a given advertisement. Currently there are 210 DMAs across the country.

We obtained advertising expenditure data on all health care providers between 2008 and 2016 and restricted the data to organizations classified as "Hospitals, Clinics & Medical Centers." Advertisers under this umbrella designation included primary care clinics, free-standing emergency rooms, specialist offices, and other types of medical-adjacent providers (laser hair removal, vein clinics etc.). To restrict the data to only acute care hospitals, we developed a multi-step strategy to match Kantar data to a CMS list of hospitals included in publicly available quality reporting documents (henceforth referred to as the master list). This process was needed to overcome several challenges inherent in matching string names across datasets using automated computer algorithms.

First, advertising may occur at the system, rather than facility-level. The master list only identifies individual hospitals and does not include any information regarding system affiliations (nor are we aware of any dataset that does). Thus, any system-level advertising captured by Kantar will not easily be matched with the corresponding hospitals through automated matching algorithms. Second, advertiser names in Kantar often include abbreviations or locally used historic names which differ from the official names used in the master data set. Third, a significant number of the non-hospital advertisers under the "Hospitals, Clinics & Medical Centers" umbrella included common words or phrases in their name which correspond to a significant number of hospitals in the master list, meaning there is a high likelihood of a false match.

We overcame these challenges in several steps. First, we eliminated any observations from the Kantar data with names that included several-hundred key words or abbreviations that would not appear in the CMS list of hospitals. This reduced both the number of potential matches and the likelihood that a hospital from the master list would be erroneously matched with a non-hospital in the Kantar data. Second, we used Stata's reclink2 command to implement a fuzzy matching algorithm between the remaining Kantar observations and the master list. We retained the best match between an advertiser and hospital in the master list based on a score which assesses the similarity between two strings or groups of strings. Approximately 20% of our matches were exactly matched based on the score. Third, we manually assessed whether all matches (even those that were exact matches) were correct based on the string name and the DMA in which the advertisement was purchased. For advertisements we determined were incorrectly matched, we used internet searches to determine whether the advertiser was a hospital or hospital system, and then identified all relevant hospitals within that system. Thus, while we used several data processing techniques to expedite matching across data sets, each advertiser included in the study's final analytic sample has been manually verified and is the result of hundreds of hours of research.

© 2021 Ndumele CD et al. *JAMA Network Open.*

eAppendix 2. Details on Composite Scores

Measure	How Measure is Constructed	Minimum Reporting Thresholds
Overall 5-Star Rating [a]	Overall star ratings are based on up to 57 measures from the following seven quality domains: patient experience, readmissions, mortality, safety of care, efficient use of medical imaging, effectiveness of care, and timeliness of care. CMS standardizes hospitals' performance on each measure by calculating Z-scores to ensure all measures are on a common scale and direction. Group scores for each domain are calculated using a latent variable model to account for the correlation between measures within a given hospital. An overall star-rating is determined by using a k-means clustering approach using the weighted average of hospitals' group scores.	For a hospital to receive a star rating, CMS sets the minimum measure threshold at three measures per group, for at least three of seven groups. At least one of the groups has to be an outcome group (e.g., mortality, safety of care, or readmissions).
CAHPS 5-Star Rating [a]	CMS averages 11 publicly reported HCAHPS star ratings to obtain a summary star rating. The constituent star-ratings measures are created by combining specific questions on the HCAHPS survey that are first converted to a 0-100 linear-scaled score. CMS adjusts scores based on patient mix, survey mode, and quarterly weighting. A clustering algorithm is used to assign stars to each quality measure, and then are averaged to determine the overall star rating.	Hospitals must have at least 100 completed HCAHPS surveys over a given four-quarter period to receive HCAHPS star ratings.
Overall Readmission Rate [a]	Medicare administrative data is used to capture unplanned readmissions that require rehospitalization within 30 days of discharge, regardless of cause. CMS reports risk standardized readmission rates by estimating hierarchical logistic regression models which adjust for selected clinical covariates and a hospital specific effect.	Hospitals must have at least 25 cases.
Mortality Composite [b]	The mortality composite combines hospital-level mortality rates for six conditions calculated by CMS: heart failure, acute myocardial infarction (AMI), pneumonia, stroke, chronic obstructive pulmonary disease, and coronary artery bypass grafting (CABG). To account for the correlation between reporting and performance on a measure, we constructed the composite as the z-score of the weighted sum of z-scores. Weights were proportional to the number of patients included in a condition-specific mortality rate, relative to the total number of patients across all conditions. We rescaled the composite by the overall mean and standard deviation of the mortality rate to facilitate interpretability.	Hospitals must have at least 25 patients with the mortality-specific condition.

Notes: [a] Scores constructed by CMS and obtained from Hospital Compare. [b] Score constructed by authors with condition-specific mortality rates obtained from Hospital Compare.

© 2021 Ndumele CD et al. *JAMA Network Open.*

eTable 1. Percentage of Advertising and Nonadvertising Hospitals (2008-2016)

	2008	2009	2010	2011	2012	2013	2014	2015	2016
Percentage of hospitals advertising	38.8	39.2	40.5	42.3	42.5	43.4	42.7	43.4	43.7
Total number of hospitals	4423	4492	4527	4550	4639	4638	4652	4617	4579
Percentage of hospitals that begin advertising who did not advertise in previous year	-	3.1	3.5	3.9	2.9	2.9	2.2	2.7	2.5
Percentage of hospitals that stop advertising who did advertise in previous year	-	2.5	1.7	2.2	1.9	1.9	2.8	2.3	2.5

© 2021 Ndumele CD et al. *JAMA Network Open.*

eTable 2. Characteristics of Advertising and Nonadvertising Hospitals
(Match within 3 years)

		No Advertising	Low Spend	Mid Spend	High Spend
**N (hospital-years)**		23,907	5,743	5,735	5,727
**Advertising Spend per Year ($)**	mean	0	13,980	73,069	504,791
	SD	0	10,280	28,781	664,628
**Net Income ($)**	mean	107,588	9,778,111	13,948,016	29,547,849
	SD	51,279,352	43,622,375	38,505,013	59,734,159
**Total Current Assets ($)**	mean	30692060	69108265	84615859	173502109
	SD	82795799	143436699	154550410	268916761
**All Inpatient Discharges**	mean	3,589.34	7,910.79	10,125.61	17,286.42
	SD	5,833.40	7,936.11	9,453.52	13,489.75
**Beds**	mean	84.53	164.85	202.85	334.27
	SD	110.10	148.69	174.67	254.24
**Bed Days Available (fully staffed)**	mean	30,334.00	59,420.01	73,547.17	121,243.6
	SD	39,979.31	53,978.81	63,435.31	92,780.07
**Occupancy**	mean	0.41	0.52	0.56	0.65
	SD	0.20	0.19	0.18	0.17
**Employees Full Time Equivalents**	mean	496.61	969.25	1,262.97	2,316.31
	SD	817.46	1,215.17	1,429.40	2,360.80
**Medicaid Inpatient Day Share[1]**	mean	0.16	0.19	0.19	0.21
	SD	0.13	0.13	0.12	0.12
**Major Teaching Hospital[2]**	mean	0.03	0.06	0.09	0.23
	SD	0.16	0.24	0.29	0.42
**Non-Profit**	mean	0.51	0.63	0.64	0.72
	SD	0.50	0.48	0.48	0.45
**For-Profit**	mean	0.20	0.24	0.25	0.17
	SD	0.40	0.43	0.43	0.37
**Public**	mean	0.29	0.13	0.11	0.12
	SD	0.46	0.33	0.31	0.32

1. Includes HMO
2. Interns and residents-to-bed > 0.25

© 2021 Ndumele CD et al. *JAMA Network Open.*

eTable 3. Adjusted Mean Differences in Performance By Year (2013-2015)

	Differences between hospitals that do and do not advertise				Differences between advertising hospitals based on intensity of advertising			
	Hospitals that do not advertise	Hospitals that do advertise	Significant differences between advertising groups (p-value) [a]		Low spend	Mid spend	High spend	Significant differences across advertising intensity groups (p-value) [b]
**2015**								
CAHPS 5-Star Rating	3.13	3.06	0.56		3.03	3.01	3.14	0.008
	(1.14)	(1.02)			(1.05)	(1.00)	(1.00)	
Mortality Composite	11.55	10.71	0.004		11.10	10.89	10.14	0.046
	(3.33)	(3.36)			(3.24)	(3.37)	(3.39)	
Overall Readmission Rate	15.19	15.3	0.34		15.22	15.30	15.39	0.67
	(0.79)	(0.96)			(0.91)	(0.89)	(1.07)	
**2014**								
Mortality Composite	12.39	11.65	0.004		12.19	11.64	11.14	0.009
	(2.98)	(2.93)			(2.81)	(2.73)	(3.14)	
Overall Readmission Rate	15.58	15.62	0.007		15.54	15.59	15.74	0.9
	(0.87)	(1.07)			(1.04)	(1.03)	(1.14)	
**2013**								
Overall Readmission Rate	15.97	16.01	0.024		15.98	15.94	16.10	0.42
	(0.92)	(1.19)			(1.19)	(1.14)	(1.23)	

Notes: Results reflect spending and performance data indicated by the year in each panel. [a] Column reflects p-value from t-test on the coefficient for a dummy variable indicating any advertising spending in a model that controls for hospital beds, net-income and census region. Ordinal logit model used for CAHPS 5-star measure and linear regression model used for mortality and readmissions measures. [b] Column reflects p-value from joint F-test of equality of spending category coefficients from model that controls for number of hospital beds, net-income, and census region. Ordinal logit model used for CAHPS 5-star measure and linear regression model used for mortality and readmissions measures. Standard deviations in parentheses.

© 2021 Ndumele CD et al. *JAMA Network Open.*

eFigure. Relationship Between Ventile of Spending and Performance Measures

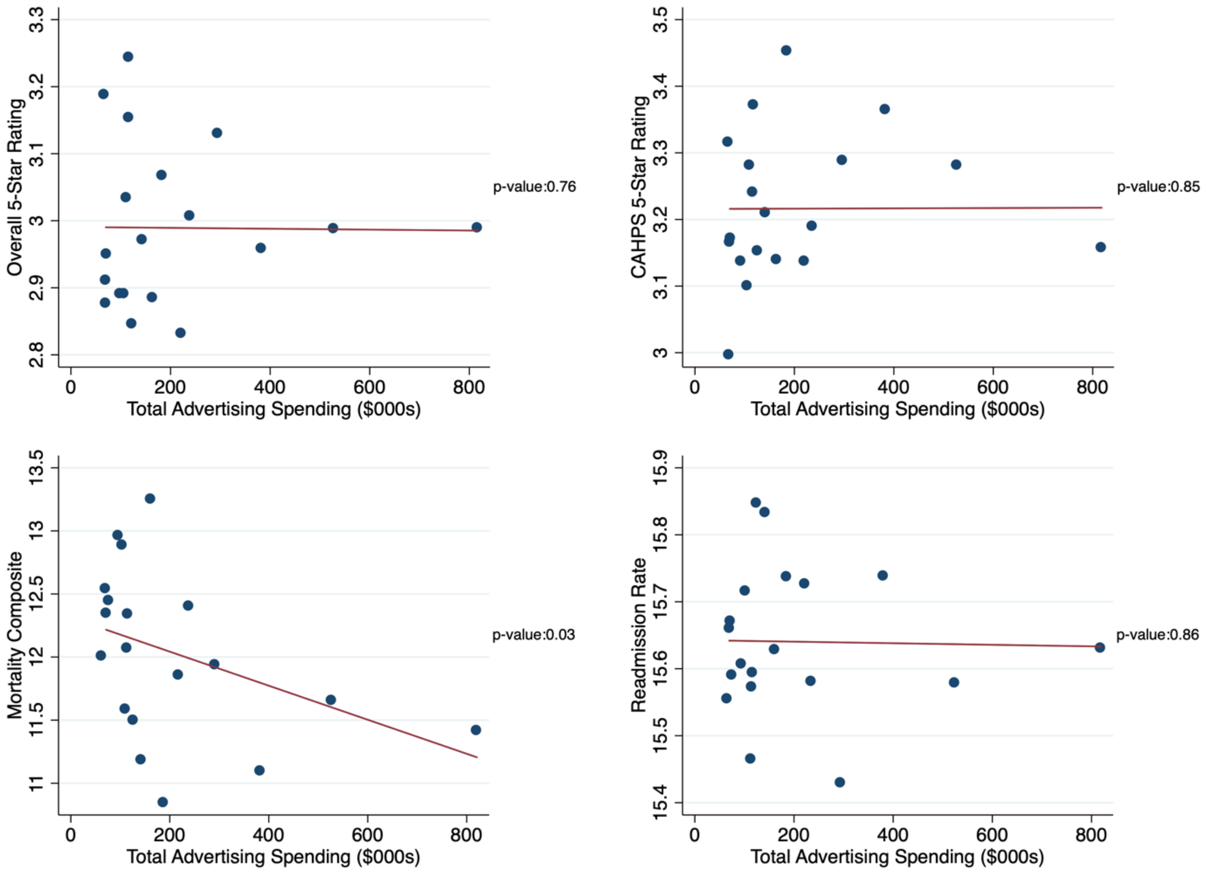

Notes: All plots reflect variables that have been residualized on number of beds, net income, and census region. Spending winsorized above $1 million dollars. P-values reflect test of the null hypothesis that the coefficient from the best fit line is equal to zero.

© 2021 Ndumele CD et al. *JAMA Network Open.*

eTable 4. Relationship Between Ad Spending and Quality Measures

	Overall 5-Star	Overall 5-Star	CAHPS 5-Star	CAHPS 5-Star	Mortality Composite	Mortality Composite	Readmission Rate	Readmission Rate
Ad Spending ($000s)	-0.000063	0.000061	-0.000033	0.000070	-0.00100***	-0.00051	0.000090*	-0.000021
	(0.000039)	(0.000044)	(0.000034)	(0.000041)	(0.00030)	(0.00026)	(0.000041)	(0.000036)
Number of Beds		-0.00090***		-0.00060***		-0.0026***		0.00071***
		(0.000095)		(0.000093)		(0.00039)		(0.000090)
Net Income		1.1e-09***		6.7e-10*		-5.0e-10		-9.5e-10***
		(2.5e-10)		(2.8e-10)		(9.6e-10)		(1.8e-10)
Constant	3.06***	2.93***	3.27***	2.98***	12.5***	11.9***	15.6***	15.8***
	(0.014)	(0.041)	(0.017)	(0.046)	(0.068)	(0.18)	(0.013)	(0.043)
Census Region Dummies	No	Yes	No	Yes	No	Yes	No	Yes

Notes: Coefficients reflect OLS regression model with advertising spending in thousands of dollars.

© 2021 Ndumele CD et al. *JAMA Network Open.*

eTable 5. Comparison of Hospital Performance Between Advertisers and Nonadvertisers Within All HRRs

All HRRs								
	**Overall 5-star rating**		**CAHPS 5-star rating**		**Mortality composite**		**Overall readmission rate**	
	N	*%*	*N*	*%*	*N*	*%*	*N*	*%*
Advertisers perform better	123	49%	119	48%	123	49%	137	54%
Non-advertisers perform better	127	51%	127	52%	129	51%	117	46%
HRRs with hospitals that do and do not advertise	250		246		252		254	
HRRs with no variation in hospital advertising	54		58		52		50	
HRRs with missing performance data	2		2		2		2	
Total	306		306		306		306	

© 2021 Ndumele CD et al. *JAMA Network Open.*

eTable 6. Comparison of Hospital Performance Between Advertisers and Nonadvertisers Within HRRs in Five or More Hospitals

HRRs with Five or More Hospitals								
	Overall 5-star rating		CAHPS 5-star rating		Mortality composite		Overall readmission rate	
	N	*%*	*N*	*%*	*N*	*%*	*N*	*%*
Advertisers perform better	113	49%	109	48%	110	48%	125	54%
Non-advertisers perform better	117	51%	117	52%	120	52%	107	46%
HRRs with hospitals that do and do not advertise	230		226		230		232	
HRRs with no variation in hospital advertising	40		44		40		38	
HRRs with missing performance data	2		2		2		2	
Total	272		272		272		272	

Notes: All percent values reflect using a denominator of HRRs with performance data.

© 2021 Ndumele CD et al. *JAMA Network Open.*

eReferences

1. Blasnik, M. "RECLINK: Stata module to probabilistically match records," Statistical Software Components (2007) S456876, Boston College Department of Economics, revised 18 Jan 2010.

2. Manning, C. D., Manning, C. D., & Schütze, H. (1999). *Foundations of statistical natural language processing*. MIT press.

3. Wasi, N., & Flaaen, A. (2015). Record Linkage Using Stata: Preprocessing, Linking, and Reviewing Utilities. *The Stata Journal*, *15*(3), 672–697. https://doi.org/10.1177/1536867X1501500304
